
JOURNAL INFORMATION
==============================
NLM Title Abbreviation: Gastroenterol Rep (Oxf)
Journal ID: Gastroenterol Rep (Oxf)
NLM Journal ID: 101620508
Journal ID: gastro

Gastroenterology Report

EISSN: 2052-0034
Publisher: Oxford University Press

ARTICLE INFORMATION
==============================
PMCID: PMC6375342
DOI: 10.1093/gastro/goz003
Article ID: goz003
Article version: 1
Subjects: Chinese Abstracts

Chinese Abstracts – Volume 7 Issue 1*

This article abstract was originally published in English. This translation has not been verified and should not be relied upon—it is provided for reference purposes only. The Publishers have not checked this translation and accept no liability for completeness or accuracy of this translation or the use of this translation for whatever purpose. This translation may be incomplete and inaccurate in whole or in part. If you need to rely upon a translation of this abstract, a professional human translator should be engaged to supply an accurate translation of the original English. When referencing articles from this journal, please always refer to the original English version, rather than a translated equivalent.

Print publication date: 2019 Feb
Electronic publication date: 2019 Feb 14
Volume: 7
Issue: 1
First page: C1
Last page: C2
Copyright: © The Author(s) 2018. Published by Oxford University Press and Sixth Affiliated Hospital of Sun Yat-Sen University
Copyright year: 2018
License: This is an Open Access article distributed under the terms of the Creative Commons Attribution License (http://creativecommons.org/licenses/by/4.0/), which permits unrestricted reuse, distribution, and reproduction in any medium, provided the original work is properly cited.
License URL: https://creativecommons.org/licenses/by/4.0/

Page count: 2

==============================
*Gastroenterology Report*, 7(1), 2019, 1–2

doi: 10.1093/gastro/goz003
Abstracts

ABSTRACTS

### Chinese Abstracts – Volume 7 Issue 1*

### Editorial （述评）

### Targeting host-microbe interaction in mucus layer: a potential treatment option for diseases
### 靶向宿主与微生物在黏液层的相互作用：一种潜在的疾病治疗方式

### Review （综述）

### Fight them or feed them: how the intestinal mucus layer manages the gut microbiota
### 肠道黏液层如何应对肠道菌群：抗击还是哺育？

肠道居住着大量微生物, 即所谓的肠道菌群。这些微生物与宿主是一种共生关系， 可协助宿主降解复杂的碳水复合物。尽管肠道菌群通常被认为是有益的， 但大量存在的微生物细胞对宿主来说也是一种永久性的威胁。因此, 肠道上皮覆以一层致密的黏液层, 以防止肠道菌群进入下层组织。肠道黏液是一种有着宿主特异性多糖结构的糖蛋白网络。黏液层长久以来一直被认为是一种被动的、宿主设计的屏障， 但最新研究显示, 黏液层的成熟和功能在很大程度上受肠道菌群的影响。反过来， 一些黏膜相关的细菌也能特异性地与黏蛋白中的多糖链结合或降解之， 将其作为营养来源。由于肠道黏液层处于宿主与微生物相互作用的关键界面, 该层结构的损坏会导致肠道菌群的侵入并最终引起炎症和感染。于是我们在结肠炎大鼠模型和结肠炎患者中， 可以观察到黏液层的功能丧失。而且， 现代社会中低纤维的西方饮食已被证实会引起细菌介导的肠道黏液层结构损害。在此， 本文对健康状态和疾病状态下肠道菌群与肠道黏液层之间的相互作用进行阐述。了解宿主与微生物之间相互作用的具体分子机制， 或可研发出针对黏液层功能失调相关疾病（如溃疡性结肠炎）新的治疗手段。

### Acute pancreatitis associated with intravenous administration of propofol: evaluation of causality in a systematic review of the literature
### 静脉注射异丙酚引起的急性胰腺炎：通过系统综述评估其因果关系

背景：异丙酚注射引起急性胰腺炎（AP）已有报道， 但两者之间的因果关系尚不明了。因此, 我们通过系统综述对其因果关系进行评估。

方法：我们于2017年8月16日对多个数据库进行检索， 由两人按预设标准对检索出的研究进行评估和筛选。使用Naranjo量表和Badalov分级评估异丙酚注射与AP的因果关系。

结果：我们筛选出来自11个国家的18项研究, 病例总数为21例, 大多数研究都有高质量的方法学。21例患者中位年龄35岁（4－77岁）, 10例（48%）为男性。8例患者注射异丙酚是用于镇静， 13例为麻醉诱导/维持， 异丙酚中位注射剂量为200 mg。14例（67%）患者具有一个中等时长（1－30天）的潜伏期（异丙酚注射至AP诊断）。4例患者甘油三酯水平>1000 mg/dL。4例（19%）患者出现严重AP。2例患者在AP治疗好转后再次接受异丙酚注射, 其中1例出现了AP复发。AP相关病死率为14%（3/21）。按Naranjo量表评估, 在19例（89%）患者中, 异丙酚注射是AP的可能原因。

结论：在Badalov Ib级别的证据支持下, 可以认为异丙酚注射是AP发生的一个可能原因。高甘油三酯血症并不是异丙酚诱发AP的唯一机制。异丙酚引起的AP中, 有19%为严重AP, 病死率高达14%。未来研究需要评估如果合并应用其他一些本身就有导致胰腺炎风险的操作（如ERCP）, 异丙酚引起的AP发生概率是否会增加。

### Consensus statement （专家共识）

### International consensus on natural orifice specimen extraction surgery (NOSES) for colorectal cancer
### 经自然腔道取标本手术（NOSES）治疗结直肠癌的国际共识

近年来， 经自然腔道取标本手术（NOSES）治疗结直肠癌已引起了广泛关注。NOSES应用于结直肠手术的潜在优势包括减轻术后疼痛， 减少伤口并发症, 减少对术后镇痛的需求, 加快肠道功能的恢复, 缩短住院时间, 更好的美容效果, 以及给患者带来了更佳的心理状态。尽管NOSES的手术创伤显著减轻， 然而该技术也存在一些潜在问题。尤其随着这项新技术的兴起， 人们对其无菌的把控、肿瘤学结果和病例选择等问题表示出关切。因此, 迫切需要一部操作指南来规范NOSES手术在结直肠癌中的开展。经过国际NOSES联盟全体成员的三轮讨论， 最终形成了该部共识。这一共识有助于NOSES在全球范围内的长远发展。

*This article abstract was originally published in English. This translation has not been verified and should not be relied upon—it is provided for reference purposes only. The Publishers have not checked this translation and accept no liability for completeness or accuracy of this translation or the use of this translation for whatever purpose. This translation may be incomplete and inaccurate in whole or in part. If you need to rely upon a translation of this abstract, a professional human translator should be engaged to supply an accurate translation of the original English. When referencing articles from this journal, please always refer to the original English version, rather than a translated equivalent.
本文摘要最初是以英文发表。中文摘译未获认证，谨供参考。出版社没有对中文摘译进行核验，不负责译文的完整性和准确性，也不会将其用以其他目的。中文摘译可能会出现部分不完整或不准确的情况。如果你需要准确获悉摘要，最好能请一位专业译者针对英文原文进行翻译。阅读杂志文章，请参考英文原文，不推荐中文翻译。

### Original article （论著）

### The physical presence of gallstone modulates *ex vivo* cholesterol crystallization pathways of human bile
### 胆石体外调节人体胆汁的胆固醇结晶途径

背景：胆固醇结晶是胆石形成的一个必要步骤。尽管一些模拟胆汁研究显示， 胆石表面与周围水相对于结晶形成过程中的胆固醇分子存在竞争关系；但相关研究尚未在人体胆汁中开展。

方法：收集13例行腹腔镜胆囊切除术患者的新鲜胆汁，其中10例为胆固醇结石， 3例为胆色素结石。再从其他2例患者收集小的胆固醇结石。应用偏光显微镜观察原始胆汁和超滤胆汁（加入或不加入胆石）中胆固醇结晶的形态，包括螺旋状和针状无水胆固醇结晶，以及片状和块状胆固醇单水结晶。分别于胆石加入胆汁前和加入21天后，称量胆石的重量。

结果：在原始胆汁中加入胆石后，无水胆固醇结晶有减少的趋势，但块状胆固醇单水结晶显著增多。在超滤胆汁中加入胆石后， 螺旋状结晶和针状结晶的形成有受抑趋势， 而片状和块状胆固醇单水结晶数量显著增多。胆汁孵育21天后，胆石重量显著减轻，无论是原始胆汁（-4.5% ± 1.6%, P = 0.046）还是超滤胆汁（-6.5% ± 1.5%, P = 0.002）。来自胆色素结石患者的胆汁，在未加入胆石前是清亮的；但加入胆固醇胆石后，所有样本均早期出现片状和块状胆固醇单水结晶。

结论：无论是在原始胆汁还是超滤胆汁中，加入胆固醇结石均可对胆固醇结晶途径产生重大影响，胆固醇单水结晶的数量增加，而无水胆固醇结晶的形成受抑。固态胆固醇结晶从胆石表面分离或可解释这一现象。

### Analysis of core protein clusters identifies candidate variable sites conferring metronidazole resistance in *Helicobacter pylori*
### 通过核心蛋白簇分析鉴定导致甲硝唑耐药的幽门螺杆菌变异位点

背景：在根治幽门螺杆菌（*Hp*）的标准三联治疗中， 甲硝唑是其中的一种一线药物。因此，甲硝唑耐药是一个重大健康问题。RdxA失活被认为是甲硝唑耐药的主要机制。然而，表达功能性RdxA蛋白的*Hp*菌珠更容易对甲硝唑耐药，提示还存在其他一些导致甲硝唑耐药的机制。

方法：我们对121份*Hp*菌珠进行了全基因组测序，其中73份是甲哨唑耐药菌珠。对包含全长RdxA基因的*Hp*临床菌珠的核心蛋白簇进行序列比对分析。对耐药菌珠与敏感菌珠的差异位点进行分析， 以鉴定出差异基因及其导致甲硝唑耐药的氨基酸改变。

结果：在73份甲硝唑耐药菌珠中， 25份（34%）是由于RdxA无义突变所导致。对来自剩余48份甲硝唑耐药菌珠和48份甲硝唑敏感菌株的核心蛋白簇进行分析发现， 有4个差异位点与甲硝唑耐药显著相关。这4个位点包括RdxA的R16H/C位点、内膜蛋白RclC（HP0565）的D85N位点、biotin carboxylase蛋白（HP0370）的V265I位点和putative threonylcarbamoyl-AMP合酶（HP0918）的A51V/T位点。

结论：我们发现了*Hp*对甲硝唑耐药的一些新的潜在机制， 值得进一步研究。

### Evaluating the effectiveness of infliximab on perianal fistulizing Crohn's disease by magnetic resonance imaging
### 通过磁共振成像评估英夫利昔单抗对肛周瘘管型克罗恩病的治疗效果

背景与目的：关于抗肿瘤坏死因子治疗肛周瘘管型克罗恩病（PFCD）影像学疗效评估的数据鲜有报道，尤其是在亚洲人群中。本研究旨在评估英夫利昔单抗（IFX）治疗PFCD的疗效， 并基于临床及影像学评估探寻“深度缓解”的预测因子。

方法：前瞻性纳入在本中心接受IFX治疗的克罗恩病合并急性肛瘘患者。分别于IFX治疗前2周和6次IFX治疗（共32周）后2周，对每例入组病例进行临床评估（瘘管流出液评估指数）、内镜评估[克罗恩病活动指数（CDAI）和肛周克罗恩病活动指数（PCDAI）]、磁共振（MRI）评估（Van Assche评分和Ng评分）以及实验室评估。

结果：在接受IFX治疗的38例入组患者中，基于瘘管流出液评估指数，52.6%获得临床缓解；基于Ng评估，42.1%获得深度缓解。在6次IFX治疗后，Van Assche评分由14.50 ± 4.26显著降低至7.36 ± 7.53, CDAI由170 ± 92显著降低至71 ± 69, PCDAI由7.45 ± 2.66显著降低至2.44 ± 3.20。多因素分析结果显示， 深度缓解的唯一预测因素是单纯性肛瘘（P = 0.004, OR = 3.802, 95% CI: 1.541－9.383）。

结论：IFX对于中国PFCD患者具有满意的疗效。MRI是评估PFCD疗效的金标准，而Van Assche评分则有一定的缺陷。

### Identification of molecular biomarkers for diagnosis of gastric cancer and lymph node metastasis
### 胃癌及其淋巴结转移的诊断学分子标志物的鉴定

背景与目的：分子标志物是肿瘤快速诊断的重要工具。本研究旨在鉴定可用于临床诊断胃癌及其淋巴结转移的分子标记物。

方法：前瞻性收集2014年12月1日至2015年12月31日期间的胃癌手术标本和相应的癌旁正常胃黏膜， 以及胃周转移性和非转移性淋巴结标本，采用实时定量PCR方法对9个目标基因进行定量检测。采用相对表达量来计算目标基因的mRNA表达水平。采用单因素方差分析（ANOVA）和Tukey多重比较检验来进行统计学比较，采用GraphPad Prism软件绘制统计分析图。

结果：在9个受检基因中， *INHBA*和*SPP1*在胃癌组织中稳定高表达，其mRNA表达水平分别是相应癌旁胃黏膜的15.4和15.6倍（P < 0.001）；前者诊断胃癌的敏感性和特异性分别为91.3%和95.7%（ROC曲线下面积0.974）， 后者的敏感性和特异性分别为82.6%和87.0%（ROC曲线下面积0.924）。进一步分析转移性与非转移性淋巴结组织中上述两个基因的表达差异，结果显示，*SPP1* mRNA表达水平两者差异无统计学意义（P = 0.470）， 而转移性淋巴结中*INHBA* mRNA表达水平上调了4.1倍（P < 0.001）， 其诊断淋巴结转移的敏感性和特异性分别为80.0%和81.5%（ROC曲线下面积0.857），而且能有效区别更严重的肿瘤进展，如T3与T4（P = 0.003）、M0与M1（P = 0.043），以及不同的组织学类型，如肠型与弥漫型（P = 0.019）。

结论：*INHBA*是用于诊断胃癌及其淋巴结转移的一种理想的分子标志物。

### Risk factors for colorectal neoplasia in patients with underlying inflammatory bowel disease: a multicenter study
### 炎症性肠病患者结直肠瘤变的危险因素：一项多中心研究

背景：本研究旨在评估中国炎症性肠病（IBD）患者肠炎相关瘤变（CAN）发生的危险因素。

方法：从8家医学中心收集1999－2016年间发生CAN的IBD患者。除了最初的病理评估， CAN的诊断尚需两位病理专家确认。最终筛选出29例CAN患者，并基于性别和IBD类型按1:3进行匹配，获得87例非CAN对照病例。

结果：29例CAN患者中，8例（27.6%）为结直肠癌，20例（69.0%）诊断为低级别上皮内瘤变，1例（3.4%）诊断为高级别上皮内瘤变。多因素分析显示，IBD诊断年龄和IBD病程是出现CAN的独立危险因素，其OR值分别为1.09（95% CI：1.04－1.14, P < 0.001）和1.14（95% CI：1.03－1.27, P = 0.013）。进一步对结直肠癌与上皮内瘤变患者进行比较，结果显示， 前者IBD诊断年龄更大（P = 0.012）， IBD病程更长（P = 0.019）。

结论：IBD诊断年龄更大、IBD病程更长者，发生CAN的概率增加。

### Case report （病例报告）

### An unusual case of gastric outlet obstruction caused by multiple giant persimmon phytobezoars
### 多块巨大柿子粪石引起的胃出口梗阻病例
